# Epigenetic age acceleration and cardiovascular outcomes in school-age children: The Generation R Study

**DOI:** 10.1186/s13148-021-01193-4

**Published:** 2021-11-16

**Authors:** Giulietta S. Monasso, Vincent W. V. Jaddoe, Leanne K. Küpers, Janine F. Felix

**Affiliations:** 1 The Generation R Study Group (Na-2918), Erasmus MC, University Medical Center Rotterdam, Rotterdam, The Netherlands; 2 Department of Pediatrics, Erasmus MC, University Medical Center Rotterdam, Rotterdam, The Netherlands; 3 Division of Human Nutrition and Health, Wageningen University, Wageningen, The Netherlands

**Keywords:** DNA methylation, Epigenetic clock, Gestational age, Cohort study, Cardiovascular disease, Carotid intima-media thickness, Atherosclerosis, Distensibility

## Abstract

**Background:**

Hypertension and atherosclerosis may partly originate in early life. Altered epigenetic aging may be a mechanism underlying associations of early-life exposures and the development of cardiovascular risk factors in childhood. A discrepancy between chronological age and age predicted from neonatal DNA methylation data is referred to as age acceleration. It may either be positive, if DNA methylation age is older than clinical age, or negative, if DNA methylation age is younger than chronological age. We examined associations of age acceleration at birth (‘gestational age acceleration’), and of age acceleration at school-age, with blood pressure and with intima-media thickness and distensibility of the common carotid artery, as markers of vascular structure and function, respectively, measured at age 10 years.

**Results:**

This study was embedded in the Generation R Study, a population-based prospective cohort study. We included 1115 children with information on cord blood DNA methylation and blood pressure, carotid intima-media thickness or carotid distensibility. Gestational age acceleration was calculated using the Bohlin epigenetic clock, which was developed specifically for cord blood DNA methylation data. It predicts gestational age based on methylation levels of 96 CpGs from HumanMethylation450 BeadChip. We observed no associations of gestational age acceleration with blood pressure, carotid intima-media thickness or carotid distensibility at age 10 years. In analyses among children with peripheral blood DNA methylation measured at age 6 (*n* = 470) and 10 (*n* = 449) years, we also observed no associations of age acceleration at these ages with the same cardiovascular outcomes, using the ‘skin and blood clock,’ which predicts age based on methylation levels at 391 CpGs from HumanMethylation450 BeadChip.

**Conclusions:**

Our findings do not provide support for the hypothesis that altered epigenetic aging during the earliest phase of life is involved in the development of cardiovascular risk factors in childhood.

**Supplementary Information:**

The online version contains supplementary material available at 10.1186/s13148-021-01193-4.

## Background

Cardiovascular risk factors such as hypertension and atherosclerosis partly originate in the earliest phases of life and track into adulthood [1–4]. Prenatal exposures, such as maternal smoking, have been associated with higher blood pressure and carotid intima-media thickness, and lower carotid distensibility in childhood [5, 6]. Arterial intima-media thickness and distensibility are related, noninvasive markers of arterial structure and function that predict cardiovascular events in adults [7, 8]. In addition to prenatal exposures, postnatal early-life exposures, such as higher weight, have been associated with suboptimal blood pressure, intima-media thickness and distensibility at school-age [9–11].

An underlying mechanism for the associations of early-life exposures with cardiovascular health later in childhood may be altered epigenetic aging [12, 13]. In recent years, multiple ‘epigenetic clocks’ have been developed [12–16]. These biomarkers estimate age from DNA methylation levels at selected cytosine-phosphate-guanine sites (CpGs). Any discrepancy between chronological age and age predicted from DNA methylation data is referred to as epigenetic age acceleration [14]. Positive age acceleration refers to an older DNA methylation age than chronological age. Negative age acceleration refers to a younger DNA methylation age than chronological age [14]. In adults, positive age acceleration has been associated with adverse outcomes, including higher risk of cancer, cardiovascular disease and all-cause mortality [14, 17, 18]. Two specific cord blood-based clocks have been developed to assess ‘gestational age acceleration’ at birth [12, 13]. Contrary to adults, there is no clear pattern in the directionality of associations of beneficial or detrimental prenatal exposures and gestational age acceleration [19]. For child ages, two clocks have been developed [20, 21]. However, in blood DNA methylation samples, these clocks were outperformed by the ‘skin and blood clock,’ which is developed among participants with a broad age range [15, 22].

Previous cross-sectional studies in adolescents and adults did not report consistently on associations of epigenetic age acceleration with blood pressure, with some studies reporting positive, and others no associations [23–28]. For aortic and carotid intima-media thickness, positive associations with epigenetic age acceleration were reported in two cross-sectional studies, one in preterm born neonates and one in adults, respectively [24, 29]. Associations with arterial distensibility have not been studied. It is not known whether age acceleration during the earliest phase of life is associated with blood pressure and carotid intima-media thickness and carotid distensibility at school-age.

We hypothesized that positive age acceleration at birth and at school-age would be associated with higher blood pressure and carotid intima-media thickness, and lower carotid distensibility at age 10 years. In a population-based study among 1115 children, we examined associations of gestational age acceleration, estimated from cord blood DNA methylation data, with blood pressure, carotid intima-media thickness and carotid distensibility at age 10 years. We also assessed associations of age acceleration at the ages of 6 and 10 years, estimated from blood DNA methylation data, with the same outcomes, among 470 and 449 children, respectively.

## Results

### Subject characteristics

In the current study, which was embedded in the Generation R Study, a prospective cohort study from pregnancy onwards, 1115 children with information on cord blood DNA methylation and at least one of the relevant outcomes at age 10 years were included for the analyses at birth [30]. We included 470 children at age 6 years and 449 children at age 10 years with information on DNA methylation measured in peripheral blood and at least one relevant outcome at age 10 years. Of these, 458 and 435 children, respectively, were also included in the analyses at birth. Additional file 1: Figure S1 shows a flowchart of the study population. Table 1 and Additional file 2: Table S1 shows subject characteristics before and after imputation for covariates, respectively. DNA methylation gestational age was estimated from cord blood DNA methylation data, using Bohlin’s epigenetic clock [13]. This clock estimates gestational age based on DNA methylation levels of 96 CpGs from HumanMethylation450 BeadChip selected trough Lasso-regression [21]. At birth, children had older clinically determined gestational age (median 40.2 weeks (95% range 37.0, 42.4)) than DNA methylation gestational age (median 39.4 weeks (95% range 37.2, 40.8)). Consequently, raw gestational age acceleration (DNA methylation age minus clinical age) had a negative median value. Pearson’s correlation coefficient between clinical gestational age and DNA methylation gestational age was 0.77 (Additional file 1: Figure S2). Among 297 children born to mothers with optimal clinical pregnancy dating, based on a regular menstrual cycle and a known first date of last menstrual period, this correlation increased slightly (*r* = 0.80). A non-response analysis suggested that as compared to children included in the analyses at birth, the 281 non-included children were more often boys and had younger, lower educated mothers, who less frequently used folic acid supplements and smoked more often during pregnancy (Additional file 2: Table S2).

**Table 1:** Characteristics of participants (*n* = 1115)^a^

*Maternal characteristics*	
Age, y	32.0 (4.0)
Educational level	
No, primary, secondary, *n* (%)	353 (32.0)
College or higher, *n* (%)	749 (68.0)
Pre-pregnancy body mass index, kg/m^2^	22.3 (18.5, 34.1)
Folic acid supplementation during pregnancy	
No supplementation, *n* (%)	62 (6.8)
Started before 10 weeks, *n* (%)	282 (31.2)
Started preconception, *n* (%)	561 (62.0)
Smoking during pregnancy	
Non-smoker or smoked until pregnancy was known, *n* (%)	900 (88.5)
Smoked throughout pregnancy, *n* (%)	117 (11.5)
Pregnancy dating based on last menstrual period	
No, *n* (%)	297 (26.6)
Yes, *n* (%)	818 (73.4)
Self-reported hypertension	
No, *n* (%)	971 (99.1)
Yes, *n* (%)	9 (0.9)
Family history of cardiovascular disease^b^	
No, *n* (%)	533 (54.2)
Yes, *n* (%)	451 (45.8)
*Birth characteristics*	
Clinical gestational age, wk	40.2 (37.0, 42.4)
DNA methylation gestational age (Bohlin), wk	39.4 (37.2, 40.8)
Raw gestational age acceleration (Bohlin), wk	− 0.90 (− 2.76, 0.92)
Residual gestational age acceleration (Bohlin), wk	0.03 (− 1.24, 1.09)
DNA methylation gestational age (Knight), wk	36.5 (32.4, 39.3)
Raw gestational age acceleration (Knight), wk	− 3.70 (− 7.44, − 1.07)
Residual gestational age acceleration (Knight), wk	0.15 (− 3.34, 2.63)
Sex	
Boy, *n* (%)	544 (48.8)
Girl, *n* (%)	571 (51.2)
*Childhood characteristics*	
At 6 y	
Age at visit^c^, y	6.0 (5.7, 7.0)
DNA methylation age (Skin and blood), y	5.6 (4.3, 7.9)
Raw age acceleration (Skin and blood), y	− 0.39 (1.7, 1.7)
Residual age acceleration (Skin and blood), y	− 0.03 (− 1.3, 2.1)
Blood pressure, mmHg	
Systolic	102 (7.7)
Diastolic	60 (6.3)
At 10 y	
Age at visit, y	
Children with DNA methylation data at birth	9.8 (9.3, 10.5)
Children with DNA methylation data at 10 y^d^	9.8 (9.2, 10.3)
DNA methylation age (Skin and blood), y	8.5 (6.7, 11.5)
Raw age acceleration (Skin and blood), y	− 1.2 (− 3.0, 1.8)
Residual age acceleration (Skin and blood), y	− 0.10 (− 1.9, 2.8)
Blood pressure, mmHg	
Systolic	103 (7.7)
Diastolic	58 (6.2)
Common carotid artery intima-media thickness, mm	0.45 (0.04)
Common carotid artery distensibility^e^, kPa^−1^*10^–3^	56.0 (37.3, 85.0)

wk, week; y, year

^a^For the analyses based on Bohlin’s epigenetic clock, we excluded 11 newborns with missing values for some of the required CpGs, leaving 1104 children for analysis in the full population and 295 children in the subgroup with optimal pregnancy dating. Values are based on observed, not imputed data and are mean (SD) or median (95% range) for continuous variables and numbers (%) for categorical variables. Missing data: maternal education: *n* = 13; maternal body mass index: *n* = 170; folic acid supplementation: *n* = 210; maternal smoking: *n* = 98

^b^We obtained this information from maternal questionnaires sent out during pregnancy. Family history of cardiovascular disease was defined as a first-degree relative with any of hypertension, myocardial infarction below the age of 65, cerebrovascular accident

^c^Of these 470 children, 12 children were not included in the analyses at birth, as they had no cord blood DNA methylation measured

^d^Of these 449 children, 14 children were not included in the analyses at birth, as they had no cord blood DNA methylation measured

^e^Indicate values before natural-log transformation

At the age of 6 years, chronological age (median 6.0; 95% range: 5.7, 7.0 years) was older than DNA methylation age (median 5.6; 95% range: 4.3, 7.9 years) estimated by the skin and blood clock (Table 1) [15]. This clock is developed on fibroblasts, keratinocytes, buccal cells, endothelial cells, blood, and saliva from newborns, children and adults and predicts age based on the methylation levels at 391 CpGs from the HumanMethylation450 BeadChip selected through elastic net regression [15]. Negative age acceleration was also observed at the age of 10 years (median chronological age 9.8; 95% range 9.2, 10.3 years; median DNA methylation age 8.5; 95% range: 6.7, 11.5; Table 1). In childhood, Pearson’s correlations between chronological age and DNA methylation age were low: *r* = 0.35 (6 years) and *r* = 0.20 (10 years) (Additional file 1: Figure S2).

### Gestational age acceleration and cardiovascular outcomes at school-age

We used linear regression models to examine the associations of gestational age acceleration with blood pressure and carotid intima-media and carotid distensibility measured at age 10 years. To examine whether the timing of outcome measurement influenced our results, we also examined associations of gestational age acceleration with blood pressure measured at age 6 years, in the subgroup of included children with these data available. In the full population, neither raw nor residual (residuals from regressing DNA methylation age on clinical age) gestational age acceleration by Bohlin’s method were associated with blood pressure, carotid intima-media thickness or carotid distensibility in the main model, which was adjusted for child sex, batch, child age at outcome measurement, cell types and maternal confounders (age, education, pre-pregnancy body mass index and both folic acid supplementation and smoking during pregnancy) (all *P* values ≥ 0.05; Tables 2 and 3). This was not different in the subgroup of 295 children born to mothers with optimal pregnancy dating. The results from basic models (not adjusted for maternal confounders) and reduced main models (not corrected for cell type proportions) are shown in Additional file 2: Table S3. Linear mixed effect models for repeated outcome measures showed that gestational age acceleration was not associated with systolic or diastolic blood pressure at school-age (Additional file 2: Table S4).

**Table 2:** Associations of gestational age acceleration by the epigenetic clock of Bohlin with blood pressure in children aged 6 years (main model)^a,b^

	Systolic blood pressure		Diastolic blood pressure	
	Difference (95% CI) in SDS	*P* value	Difference (95% CI) in SDS	*P* value
Full population (*n* = 1104)	*n* = 994		*n* = 994	
Raw	0.056 (− 0.01, 0.13)	0.12	− 0.001 (− 0.07, 0.07)	0.98
Residual	0.011 (− 0.11, 0.13)	0.85	0.033 (− 0.15, 0.08)	0.58
Subgroup: optimal pregnancy dating (n = 295)	*n* = 264		*n* = 264	
Raw	− 0.033 (− 0.17, 0.11)	0.64	− 0.060 (− 0.20, 0.08)	0.41
Residual	− 0.123 (− 0.36, 0.12)	0.31	− 0.016 (− 0.26, 0.23)	0.89

Values represent regression coefficients (95% confidence interval) and reflect the difference in blood pressure in SDS per change in raw and residual gestational age acceleration (in weeks) at birth. Shown results are based on the main model which was adjusted for child sex, batch effects in DNA methylation data (by including sample plate number), child age at outcome measurement, cell types and maternal confounders (age, education, pre-pregnancy body mass index and folic acid supplementation and smoking during pregnancy)

CI, confidence interval; SDS, standard deviation score

^a^For the analyses based on Bohlin’s epigenetic clock, we excluded 11 of the 1115 included newborns with missing values for some of the required CpGs, leaving 1104 children for analysis in the full population. The subgroup included children born to mothers with optimal pregnancy dating based on a regular menstrual cycle and gestational age determined by last menstrual period. For the analyses based on Bohlin’s epigenetic clock, we excluded 2 of 297 included newborns with missing values for some of the required CpGs, leaving 295 children for analysis

^b^Raw gestational age acceleration was obtained by subtracting the clinical estimate of gestational age from DNA methylation gestational age. Residual gestational age acceleration was calculated from the residuals from a regression model of DNA methylation gestational age on clinical gestational age

**Table 3:** Associations of gestational age acceleration by the epigenetic clock of Bohlin with cardiovascular outcomes in children aged 10 years (main model)^a,b^

	Systolic blood pressure		Diastolic blood pressure		Common carotid artery intima-media thickness		Common carotid artery distensibility	
	Difference (95% CI) in SDS	*P* value	Difference (95% CI) in SDS	*P* value	Difference (95% CI) in SDS	*P* value	Difference (95% CI) in SDS	*P* value
Full population (*n* = 1104)	*n* = 1097		*n* = 1098		*n* = 1060		*n* = 943	
Raw	0.041 (− 0.02, 0.11)	0.21	0.021 (− 0.05, 0.09)	0.54	0.002 (− 0.06, 0.07)	0.96	0.001 (− 0.07, 0.07)	0.97
Residual	− 0.030 (− 0.14, 0.08)	0.60	0.011 (− 0.10, 0.12)	0.82	0.037 (− 0.07, 0.15)	0.52	0.068 (− 0.05, 0.19)	0.26
Subgroup: optimal pregnancy dating (*n* = 295)	*n* = 293		*n* = 293		*n* = 280		*n* = 255	
Raw	0.049 (− 0.09, 0.19)	0.49	0.026 (− 0.11, 0.17)	0.72	0.073 (− 0.06, 0.21)	0.29	− 0.054 (− 0.20, 0.09)	0.47
Residual	− 0.161 (− 0.40, 0.08)	0.18	− 0.032 (− 0.27, 0.21)	0.79	0.146 (− 0.09, 0.38)	0.22	0.174 (− 0.07, 0.42)	0.16

Values represent regression coefficients (95% confidence interval) and reflect the difference in cardiovascular outcome in SDS per change in raw and residual gestational age acceleration (in weeks) at birth. Shown results are based on the main model which was adjusted for child sex, batch effects in DNA methylation data (by including sample plate number), child age at outcome measurement, cell types and maternal confounders (age, education, pre-pregnancy body mass index and folic acid supplementation and smoking during pregnancy)

CI, confidence interval; SDS, standard deviation score

^a^For the analyses based on Bohlin’s epigenetic clock, we excluded 11 of the 1115 included newborns with missing values for some of the required CpGs, leaving 1104 children for analysis in the full population. The subgroup included children born to mothers with optimal pregnancy dating based on a regular menstrual cycle and gestational age determined by last menstrual period. For the analyses based on Bohlin’s epigenetic clock, we excluded 2 of 297 included newborns with missing values for some of the required CpGs, leaving 295 children for analysis

^b^Raw gestational age acceleration was obtained by subtracting the clinical estimate of gestational age from DNA methylation gestational age. Residual gestational age acceleration was calculated from the residuals from a regression model of DNA methylation gestational age on clinical gestational age

A sensitivity analysis showed that results obtained for the right and left common carotid artery separately were similar, indicating that there were no differences based on the side of carotid artery measurement (Additional file 2: Table S5). We also estimated gestational age acceleration using another cord blood-based epigenetic clock, developed by Knight. This clock estimates gestational age based on methylation levels of 148 CpGs from the HumanMethylation27 BeadChip selected through elastic net regression [12]. Pearson’s correlation coefficients between clinical gestational age and DNA methylation gestational age by Knight in the full population and subgroup with optimal pregnancy dating are shown in Additional file 1: Figure S2. We also observed no significant associations of gestational age acceleration by Knight with blood pressure, carotid intima-media thickness or carotid distensibility at age 10 years (Additional file 2: Tables S6 and S7).

### Childhood age acceleration and cardiovascular outcomes at school-age

Age acceleration estimated by the ‘skin and blood’ clock at ages 6 and 10 years was not associated with blood pressure at either age. Similarly, it was not associated with carotid intima-media thickness and carotid distensibility at age 10 years (main model, Tables 4, 5) [15]. Conditional regression analyses that accounted for the correlations between repeated age acceleration measurements showed that age acceleration at age 6 years was not associated with systolic or diastolic blood pressure, carotid intima-media thickness or carotid distensibility at age 10 years. Also, independent of epigenetic age acceleration at age 6 years, age acceleration at age 10 years was not associated with these cardiovascular outcomes (Additional file 2: Table S8). In neither the analyses at birth nor the analyses at school-age did the results change substantially upon additional adjustment for maternal history of hypertension and family history of cardiovascular disease (data not shown).

**Table 4:** Associations of childhood age acceleration based on the skin and blood clock with blood pressure in children aged 6 years (main model)^a^

	Systolic blood pressure (*n* = 438)		Diastolic blood pressure (*n* = 438)	
	Difference (95% CI) in SDS	*P* value	Difference (95% CI) in SDS	*P* value
Raw	0.067 (− 0.08, 0.21)	0.35	0.062 (− 0.08, 0.20)	0.39
Residual	0.067 (− 0.08, 0.21)	0.35	0.062 (− 0.08, 0.20)	0.39

Values represent regression coefficients (95% confidence interval) and reflect the difference in blood pressure in SDS per change in raw and residual age acceleration (in weeks). Shown results are based on the main models which were adjusted for child sex, batch effects (by including plate number), child age at outcome measurement, cell types and maternal confounders (age, education, pre-pregnancy body mass index and folic acid supplementation and smoking during pregnancy)

CI, confidence interval; SDS, standard deviation score

^a^Raw age acceleration was obtained by subtracting the clinical estimate of age from DNA methylation age. Residual age acceleration was calculated from the residuals from a regression model of DNA methylation age on clinical age. The correlation between raw and residual age acceleration was almost perfect (*r* = 0.996)

**Table 5:** Associations of childhood age acceleration based on the skin and blood clock with cardiovascular outcomes in children aged 10 years (main model)^a^

		Systolic blood pressure		Diastolic blood pressure		Common carotid artery intima-media thickness		Common carotid artery distensibility	
		Difference (95% CI) in SDS	*P* value	Difference (95% CI) in SDS	*P* value	Difference (95% CI) in SDS	*P* value	Difference (95% CI) in SDS	*P* value
Six years (*n* = 470)		*n* = 468		*n* = 468		*n* = 452		*n* = 412	
Raw		− 0.020 (− 0.15, 0.11)	0.77	0.033 (− 0.10, 0.17)	0.64	− 0.015 (− 0.15, 0.12)	0.83	− 0.001 (− 0.14, 0.14)	0.99
Residual		− 0.024 (− 0.16, 0.11)	0.73	0.045 (− 0.09, 0.18)	0.52	0.001 (− 0.14, 0.14)	0.99	− 0.009 (− 0.15, 0.13)	0.90
Ten years (*n* = 449)		*n* = 448		*n* = 448		*n* = 432		*n* = 375	
Raw		0.032 (− 0.06, 0.12)	0.49	0.034 (− 0.06, 0.13)	0.46	− 0.018 (− 0.11, 0.07)	0.70	− 0.034 (− 0.14, 0.07)	0.51
Residual		0.032 (− 0.06, 0.12)	0.49	0.034 (− 0.06, 0.13)	0.46	− 0.018 (− 0.11, 0.07)	0.70	− 0.034 (− 0.14, 0.07)	0.51

Values represent regression coefficients (95% confidence interval) and reflect the difference in cardiovascular outcome in SDS per change in raw and residual age acceleration (in weeks). Shown results are based on the main models which were adjusted for child sex, batch effects (by including plate number), child age at outcome measurement, cell types and maternal confounders (age, education, pre-pregnancy body mass index and folic acid supplementation and smoking during pregnancy)

CI, confidence interval; SDS, standard deviation score

^a^Raw age acceleration was obtained by subtracting the clinical estimate of age from DNA methylation age. Residual age acceleration was calculated from the residuals from a regression model of DNA methylation age on clinical age. In childhood, the correlation between raw and residual age acceleration was almost perfect (*r* = 0.996 and *r* = 0.999 for 6 and 10 years, respectively)

## Discussion

In this population-based prospective cohort study, we observed that age acceleration at birth or in childhood is not associated with blood pressure, carotid intima-media thickness and carotid distensibility at school-age. These correlated cardiovascular risk factors partly originate in the earliest phase of life [1–6, 9–11]. Altered epigenetic aging may be an underlying mechanism for associations of early-life exposures with cardiovascular health at school-age [12–16, 19]. We hypothesized that positive epigenetic age acceleration at birth and at school-age would be associated with higher blood pressure and carotid intima-media thickness, and lower carotid distensibility at the age of 10 years.

We observed no associations of gestational age acceleration with blood pressure and carotid intima-media thickness and carotid distensibility in childhood. These null findings were not explained by inaccurate clinical pregnancy dating, as we obtained similar results among children born to mothers with a regular menstrual cycle and pregnancy dating based on last menstrual period. Previous cross-sectional studies reported inconsistently on associations of epigenetic age acceleration with blood pressure [23–28, 31, 32]. Both an American and a Scottish study among 969 and 5100 adults, respectively, reported that positive age acceleration as estimated by the clock of Hannum, but not that of Horvath, was associated with hypertension [26, 28]. A study among 1100 mostly hypertensive African-Americans reported that positive age acceleration was associated with blood pressure, using Horvath’s and Hannum’s clocks [27]. In addition, this study, as well as two studies among 4178 women from the Women’s Health Initiative, only reported associations with systolic but not diastolic blood pressure when using the PhenoAge and GrimAge clocks [27, 31, 32]. Three further studies in adults and one in adolescents reported no associations of age acceleration with blood pressure [23–25, 33].

For intima-media thickness, an Australian study among 169 neonates reported that gestational age acceleration estimated in saliva using the clock of Horvath was positively associated with aortic intima-media thickness measured within 24 h postpartum in preterm, but not term, born neonates [29]. Another cross-sectional study, among 2543 middle-aged African-Americans, reported positive associations of age acceleration estimated by both the clocks of Hannum and Horvath with carotid intima-media thickness [24]. Associations of age acceleration with arterial distensibility have not been studied, to the best of our knowledge. Overall, previous cross-sectional studies show some evidence for positive associations of age acceleration with blood pressure and carotid intima-media thickness. Results from our population-based cohort study, however, suggest no prospective or cross-sectional associations of age acceleration with these outcomes, nor with carotid distensibility, at school-age.

Our null findings may be interpreted in two ways. First, it could be that DNA methylation is simply not a mechanism underlying the associations of early-life exposures with childhood health. Some exposures, such as maternal smoking and folate concentrations during pregnancy, have been related to both differential DNA methylation and childhood cardiovascular health [5, 34–36]. This does not necessarily imply that DNA methylation is on the biological pathway between these exposures and childhood cardiovascular health. DNA methylation changes could be epiphenomena rather than consequences of early-life exposures [37]. Second, DNA methylation may in fact be on the causal pathway from early-life exposures to childhood cardiovascular health, but at specific CpG sites that are not captured by the three clocks we used in this study. Only one CpG (cg07141002) and three CpGs (cg01243072, cg06690548, cg18933331) included in the clock of Knight and the skin and blood clock, respectively, have been individually associated with blood pressure in adults [38, 39]. The only CpG (cg05575921) that has been associated with carotid intima-media thickness in adults is not included in any of the three clocks, whereas arterial distensibility has not been studied in relation to DNA methylation [38, 40]. In adults, different epigenetic clocks seem to capture different age-related biological processes [16]. In addition, previous studies in newborns and adults observed different associations of age acceleration with cardiometabolic outcomes, depending on which clock was used [26–28, 41, 42]. The clocks of Bohlin and Knight and the skin and blood clock may thus capture aspects of age acceleration that are not a reflection of cardiovascular adaptations in utero or in childhood.

The null findings may also be explained by some limitations of our study. First, although we had a relatively large study population, we may have been underpowered to find associations of smaller magnitude, especially in the subgroup with optimal pregnancy dating. Although the correlation between clinical gestational age and DNA methylation gestational age increased in this subgroup, the decrease in power may not have been outweighed by increased precision in the estimation of clinical gestational age. Second, our study population was relatively healthy. Limited variation in either age acceleration or in the outcomes may have prevented detection of meaningful associations. It needs confirmation whether associations are observed among populations showing more extreme variation in exposure or outcomes. Despite these limitations, our study also had strengths. These include its implementation in a large observational prospective birth cohort, the availability of repeated DNA methylation measurements and detailed cardiovascular outcomes, and the replication of the null findings of the main analysis at birth using another epigenetic clock.

## Conclusions

Epigenetic age acceleration at birth or at school-age was not associated with blood pressure at ages 6 and 10 years or with carotid intima-media thickness and carotid distensibility at age 10 years. Our study does not support altered epigenetic aging as an underlying mechanism for associations of exposures during the earliest phase of life with childhood cardiovascular health. Further research is needed to confirm this.

## Methods

### Participants

This study was embedded in the Generation R Study, a population-based prospective cohort study from fetal life onwards in Rotterdam, the Netherlands [30]. The Medical Ethical Committee of Erasmus MC, University Medical Center Rotterdam, approved the study (MEC 198.782/2001/31). Pregnant women with an expected delivery date between April 2002 and January 2006 living in Rotterdam were eligible to participate. We obtained written informed consent from all participants. In a relatively homogeneous European-ancestry subgroup taken from the total of 9901 live-born participating children, we measured genome-wide DNA methylation in cord blood taken at birth (*n* = 1396) and in peripheral blood at 6 years (*n* = 493) and 10 years (*n* = 464), when the children visited the research center for follow-up visits. Additionally, at age 10 years, we measured systolic and diastolic blood pressure and both common carotid artery intima-media thickness and carotid distensibility. In the current study, we included children with DNA methylation measured at any time point, and at least one of these cardiovascular outcomes measured (Additional file 1: Figure S1). There were no twins among our population for analysis. For each sibling pair, we included only one child by selecting based on completeness of outcome data, and if equal, randomly.

### DNA methylation data

We used the salting-out method to extract DNA from cord blood and peripheral blood samples in childhood. Five-hundred nanograms of DNA were bisulfite converted using the EZ-96 DNA Methylation kit (Shallow) (Zymo Research Corporation, Irvine, USA). DNA methylation samples were plated onto 96-well plates in no specific order. Samples were processed with the Illumina Infinium HumanMethylation450 BeadChip (Illumina Inc., San Diego, USA). Quality control and normalization were performed using the CPACOR workflow [43]. Probes with a detection *P* value ≥ 1E-16 were set to missing. Intensity values were quantile normalized. We removed arrays with technical problems, a call rate ≤ 95%, or a mismatch between the expected sex of participant and sex determined by chromosome X and Y probe intensities. Probes on the sex chromosomes were removed before the analyses. We used untransformed beta-values as measures of DNA methylation. The final DNA methylation dataset contained information on 458,563 CpGs.

### Gestational age estimation

Mothers of participating children visited the research center in the first trimester of the index pregnancy for fetal ultrasound. During this visit, we established the ‘clinical’ estimate of gestational age [44]. For those mothers with a known and reliable first day of the last menstrual period and a regular (28 ± 4 days) menstrual cycle, gestational age was based on last menstrual period [44]. If mothers did not know their last menstrual period exactly, or had an irregular menstrual cycle, we established gestational age by ultrasound examination. Gestational age at birth was assessed from midwife or obstetric records. We calculated DNA methylation gestational age at birth (in weeks) using the epigenetic clock of Bohlin as primary method, as it was developed among newborns comparable to those in our study population [13]. This clock estimates gestational age from DNA methylation levels at 96 CpGs from HumanMethylation450 BeadChip selected trough Lasso-regression [21]. Bohlin’s DNA methylation gestational age was calculated using the GAprediction package version 1.16.0 in R 3.6.1 [13, 45]. We excluded children with missing values for one or more of the 96 required CpGs (total study population: *n* = 11; subgroup with optimal pregnancy dating: *n* = 2). As a sensitivity analysis, to examine the robustness of our findings, we estimated DNA methylation gestational age by another cord blood based epigenetic clock, developed by Knight [12]. This clock estimates gestational age from DNA methylation levels at 148 CpGs that are available on the HumanMethylation450 BeadChip, selected through elastic net regression [12, 46]. Knight’s DNA methylation gestational age was calculated using the methylclock package 0.5.0 in R 3.6.1 (R Core Team, Vienna, Austria) [47]. All children had information on the 148 required CpGs.

### Childhood age estimation

Of the 470 and 449 children included in the analyses at age 6 and age 10 years, respectively, twelve (6 years) and fourteen (10 years) children were not included in the analyses at birth. Childhood DNA methylation age (in years) was estimated by the ‘skin and blood clock’, using the methylclock package 0.5.0 in R 3.6.1 (R Core Team, Vienna, Austria) [15, 47]. A previous study reported that this well-established clock outperformed two child-specific clocks when estimating DNA methylation age in blood [20–22]. The skin and blood clock estimates age from DNA methylation levels at 391 CpGs measured in fibroblasts, keratinocytes, buccal cells, endothelial cells, blood, and saliva from participants aged 0–94 years [15]. These CpGs were automatically selected trough elastic net regression that were available on the HumanMethylation450 BeadChip [15]. We obtained chronological (‘clinical’) age (in years) at the time of the child’s follow-up visit to the research center.

### Age acceleration at birth and in childhood

Based on the epigenetic clocks of Bohlin and Knight at birth and the skin and blood clock at school-age, we calculated both raw and residual age acceleration, in line with previous research [46–48]. Raw age acceleration was obtained by subtracting clinical age from DNA methylation age. It does not take into account the potential confounding effect of clinical age on DNA methylation age, as variance is shared between both measures [46]. We obtained residual age acceleration from the residuals from a linear regression of DNA methylation age on clinical age. By definition, residual acceleration is uncorrelated with clinical age [46]. Positive age acceleration indicates older DNA methylation age than clinical age. Negative age acceleration indicates younger DNA methylation age than clinical age.

### Cardiovascular outcomes at school-age

When children visited the research facility at age 6 and 10 years, we assessed blood pressure at the right brachial artery four times with one minute intervals using the validated automatic sphygmomanometer Datascope Accutorr Plus (Paramus, New Jersey, United States) [49]. For children with complete data on blood pressure measurements available, the mean systolic and diastolic blood pressure was calculated from the last three measurements. Further, during the visit at age 10 years, we measured carotid intima-media thickness and carotid distensibility three times at both common carotid arteries (*n* = 5,746) using the Logiq E9 (GE Medical Systems, Wauwatosa, WI, USA) device. Children were in the supine position, with the head tilted slightly away from the transducer. The common carotid artery was identified in a longitudinal plane, ~ 10 mm proximal from the carotid bifurcation. We obtained six recordings that each ideally included multiple heart cycles. The analyses were performed offline and semi-automatically, using the application Carotid Studio (Cardiovascular Suite (Quipu srl, Pisa, Italy)). For each recording, at all R-waves of the simultaneous ECG, carotid intima-media thickness was computed at the far wall, as the average distance between lumen-intima and media-adventitia borders. The average carotid intima-media thickness of all frames of the acquired image sequence was computed. Carotid distensibility was defined as the relative change in lumen area during systole for a given peripheral brachial artery pressure change. The lumen diameter of the carotid artery was computed as the average distance between the far and near media‐adventitia interfaces, for each frame of the acquired image sequence. Distension was calculated as the difference between maximal (diastolic) and minimal (systolic) lumen diameter. Per recording, the average distension and diameter values were used to compute the average carotid distensibility. During these offline analyses, we excluded 516 and 704 children, without any valid carotid intima-media thickness or carotid distensibility measurement, respectively. Reasons were no appropriate recording, insufficient quality of the recording, recording of the heart only, or no blood pressure measurement available to calculate carotid distensibility. Further data processing for the remaining 5,230 and 5,042 children with carotid intima-media thickness and carotid distensibility data, respectively, was performed using R (R Core Team, Vienna, Austria). We excluded 9 children with unreliable low or high carotid distensibility values. We calculated the overall mean carotid intima-media thickness (in mm) and carotid distensibility (in kPa^−1^*10^–3^), both based on up to six measurements. In a reproducibility study performed among 47 subjects, the interobserver and intraobserver intraclass correlation coefficient were > 0.85 for carotid distensibility and > 0.94 for carotid intima-media thickness.

### Covariates

We selected potential covariates based on the previous literature. From maternal questionnaires sent out at intake, we obtained information on the following maternal covariates: age (in years), educational level (primary or secondary education, versus college or higher), pre-pregnancy body mass index (kg/m^2^) and both folic acid supplementation (no supplementation, started before ten weeks gestational age and started preconception) and smoking (no smoking or quit before second trimester, versus sustained smoking) during pregnancy. We also obtained information on maternal hypertension and family history of cardiovascular disease, which we defined as hypertension, myocardial infarction before the age of 65, or cerebrovascular attack in a first-degree relative. From midwife and medical records, we obtained information on child sex. We adjusted for batch effects to correct for systematic technical variation in the DNA methylation data by including sample plate number. For the analyses of gestational age acceleration, we used the “Salas” reference set to estimate cell type proportion in the ‘FlowSorted.CordBlood.Combined.450 K’ Bioconductor package. It includes CD8+ T cells, CD4+ T cells, Natural Killer cells, B cells, Monocytes, Granulocytes and Nucleated red blood cells [50]. For the analyses in childhood, we used the Houseman method based on the Reinius reference set for cell type estimation (CD8+ T-cells, CD4+ T-cells, Natural Killer cells, B cells, Monocytes, Granulocytes) in the ‘FlowSorted.Blood.450 K’ package [51, 52].

### Statistical analysis

First, we performed a non-response analysis using Student’s *t*-tests, Mann–Whitney tests and Chi-square tests. Among those children with cord blood DNA methylation measured, we compared characteristics of children included in the analyses at birth, to those of non-included children because they had no information on any cardiovascular outcome of interest, or had a sibling included in the analysis. We used multiple imputations for covariates with missing values, using the Markov Chain Monte Carlo method. We created five datasets and pooled analysis estimates from all datasets [53]. Second, we examined the associations of raw and residual gestational age acceleration estimated by both the method of Bohlin, with all cardiovascular outcomes, using multivariable linear regression models. To compare effect estimates, the outcomes were analyzed in SDS, after natural log transformation of carotid distensibility, which had a skewed distribution. Basic models were adjusted for sample plate number (batch), estimated cell types, child sex and age at outcome measurement. Main models were additionally adjusted for maternal age, education, pre-pregnancy body mass index, folic acid supplementation and smoking. In line with previous research, the main model was also run without cell type adjustment (reduced main model), to examine the specific influence of variation in cell type proportions [54]. We performed three sensitivity analyses. First, in an attempt to minimize the chance of measurement error in the calculation of gestational age acceleration, we restricted the analyses at birth to children of mothers with optimal pregnancy dating, based on last menstrual period. We reasoned that such error may result from inaccuracy in the prediction of clinical gestational age by fetal ultrasound, which does not take into account variation in early fetal growth [42]. Second, as recommended in previous literature, we assessed the associations of gestational age acceleration with carotid intima-media thickness and carotid distensibility measured at the right versus left common carotid artery [55]. Third, both in the full population and in the subgroup with optimal pregnancy dating, we reran the main model after calculating gestational age acceleration based on the clock of Knight. In childhood, we assessed the associations of raw and residual age acceleration at age 6 and 10 years with all outcomes (main models only). Further, we explored whether the timing of outcome measurement is critical for associations of age acceleration with blood pressure, on which we had information at age 6 years. To do this, we analyzed associations of age acceleration at birth and at 6 years with systolic and diastolic blood pressure. Finally, as a secondary analysis, in the full population and for each time point and all outcomes, we additionally adjusted the main model for both maternal hypertension and family history of cardiovascular disease. We considered *P* values < (0.05/4 assessed outcomes), so < 0.00125 statistically significant. We did not stratify the analyses on sex because the interaction terms between age acceleration measures and sex were not significant. We examined potential nonlinear associations between gestational age acceleration and all outcomes by adding a quadratic term for raw or residual gestational age acceleration to the main models. None of the quadratic terms were significant (all *P* > 0.05). All statistical analyses were performed using the Statistical Package of Social Sciences version 25.0 for Windows (SPSS IBM, Chicago, Illinois, United States).

### Longitudinal analyses

Making use of the repeated measurements in our data, we performed linear mixed effects models using the R package nlme to examine the associations of gestational age acceleration with repeatedly measured systolic and diastolic blood pressure at ages 6 and 10 years. To decrease the number of covariates, we first obtained standardized residuals for both raw and residual gestational age acceleration from the regression of gestational age acceleration on batch and all cell types and used these residuals as the exposure. All models included a random intercept. We additionally included an interaction term between age acceleration and child age to allow the exposure-outcome association to change across childhood. We also examined the independent associations of age acceleration at ages 6 and 10 years with all cardiovascular outcomes at age 10 years using conditional multivariable linear regression to account for the correlations between epigenetic age measurements using SPSS [56]. For these analyses, only the 329 children with information on age acceleration at both age 6 and age 10 years were included. First, for both ages, we obtained standardized residuals for both raw and residual age acceleration from the regression of age acceleration on batch and all cell types. Second, the obtained residuals at 10 years were made independent of those at 6 years by regressing the 10-year residuals on those at 6 years. Third, both measures were simultaneously included in the models.

## Supplementary Information

**Additional file 1. Figure S1**. Flowchart of the study population. **Figure S2**. Pearson’s correlation between clinical age and DNA methylation age.

**Additional file 2. Table S1**. Maternal and child characteristics based on imputed data (*n* = 1115). **Table S2**. Non-response analysis. **Table S3**. Associations of gestational age acceleration by the epigenetic clock of Bohlin with cardiovascular outcomes in children aged ten years (basic model and reduced main model). **Table S4**. Associations of gestational age acceleration by the epigenetic clock of Bohlin with repeated blood pressure measurements (linear mixed effect models). **Table S5**. Associations of gestational age acceleration by the epigenetic clock of Bohlin with carotid intima-media thickness and carotid distensibility in children aged ten years, for the right and left common carotid artery separately (main model). **Table S6**. Associations of gestational age acceleration by the epigenetic clock of Knight with blood pressure in children aged six years (main model). **Table S7**. Associations of gestational age acceleration by the epigenetic clock of Knight with cardiovascular outcomes in children aged ten years (main model). **Table S8**. Associations of repeated epigenetic age acceleration measurements at age 6 and 10 years with cardiovascular outcomes in children aged 10 years (conditional regression analyses)

## Data Availability

The datasets generated and/or analyzed during the current study are not publicly available due to privacy restrictions, but are available from the corresponding author on reasonable request, subject to the Generation R Study data access procedures.

## Abbreviations

CI: Confidence interval
CpG: Cytosine-guanine dinucleotide
CpGs: CpG sites
EWAS: Epigenome-wide association study
SD: Standard deviation
SDS: Standard deviation score
