# Supplementary material for: Epigenetic age acceleration and cardiovascular outcomes in school-age children: The Generation R Study
Source: Clin Epigenetics. 2021 Nov 16;13:205. doi: 10.1186/s13148-021-01193-4 (PMC8597298; doi:10.1186/s13148-021-01193-4)
Supplement: Supplementary file 1 — Additional file 1. Figure S1. Flowchart of the study population. Figure S2. Pearson’s correlation between clinical age and DNA methylation age. [file 13148_2021_1193_MOESM1_ESM.docx]

**Additional file 1**

**Epigenetic age acceleration and cardiovascular outcomes in school-age children: the Generation R Study**

Giulietta S. Monasso^1,2^, Vincent W.V. Jaddoe^1,2^, Leanne K. Küpers^1,2,3^, Janine F. Felix^1,2^

1. The Generation R Study Group, Erasmus MC, University Medical Center Rotterdam, Rotterdam, the Netherlands
2. Department of Pediatrics, Erasmus MC, University Medical Center Rotterdam, Rotterdam, the Netherlands
3. Division of Human Nutrition and Health, Wageningen University, Wageningen, The Netherlands

**Content: 2 Figures**

**Address for correspondence:** Janine F. Felix, MD PhD, Generation R Study Group (Na-2918), Erasmus MC, University Medical Center Rotterdam, Rotterdam, the Netherlands. Phone: +31 10 7043405, fax: +31 10 70 44645, email: j.felix@erasmusmc.nl

Children participating in the Generation R Study with DNA methylation measured in cord blood or peripheral blood in childhood

Birth: n=1396

Six year: n=493

Ten year: n=464

Exclusion: Children without data on blood pressure, common carotid artery intima-media thickness or distensibility at age ten years

Birth: n=269

Six year: n=19

Ten year: n=13

Exclusion: One of each sibling pair

Birth: n=12

Six year: n=4

Ten year: n=2

Final populations for analysis after imputation for covariates

| **Birth** | |  | | |
| --- | --- | --- | --- | --- |
| All children n=1115^a,b^ | | Subgroup with optimal pregnancy dating n=297^c^ | | |
| Systolic blood pressure: | | Systolic blood pressure | | |
| At six years n=1005 | | At six years n=266 | | |
| At ten years n=1108 | | At ten years n= 295 | | |
| Diastolic blood pressure: | | Diastolic blood pressure | | |
| At six years n=1005 | | At six years n=266 | | |
| At ten years n=1109 | | At ten years n= 295 | | |
| Intima-media thickness: n=1071 | | Intima-media thickness: n=282 | | |
| Distensibility: n=954 | | Distensibility: n=257 | | |
| **Six year** | |  | | |
| All children n=470^d^ | |  | | |
| Systolic blood pressure: | | |  | |
| At six years n=438 | | |  | |
| At ten years n=468 | | |  | |
| Diastolic blood pressure: | | |  | |
| At six years n=438 | | |  | |
| At ten years n=468 | | |  | |
| Intima-media thickness: n=452 | | |  | |
| Distensibility: n=412 | | |  | |
| **Ten year** | |  | | |
| All children n=449^e^ | |  | | |
| Systolic blood pressure: n=448 | | |  | |
| Diastolic blood pressure: n=448 | | |  | |
| Intima-media thickness: n=432 | | |  | |
| Distensibility: n=375 | | |  | |

**Figure S1. Flow chart of the study population**

a The non-response analysis compared the children who were included in the analyses to those children who also had information on cord blood DNA methylation, but either had no information on any cardiovascular outcome or had a sibling that participated in the analyses (n=281).

b For the analyses based on Bohlin’s epigenetic clock, we excluded 11 newborns with missing values for some of the required CpGs, leaving 1104 children for analysis in the full population and 295 children in the subgroup with optimal pregnancy dating.

c An optimal pregnancy dating was based on regular menstrual cycle of 28 ± 4 days and a known date of last menstrual period. Because of low numbers, this sensitivity analysis was not performed for the analyses in childhood.

d Of these, 12 children were not included in the analyses at birth as they had no cord blood DNA methylation measured.

e Of these, 14 children were not included in the analyses at birth as they had no cord blood DNA methylation measured.

**A B**


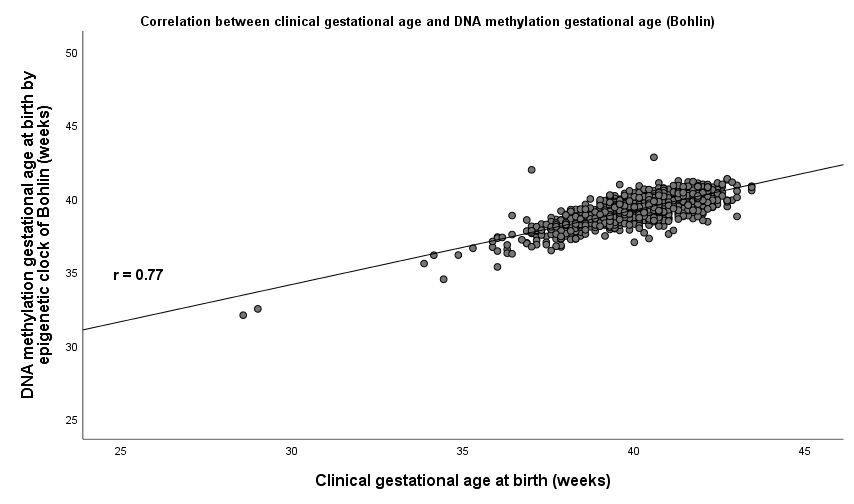

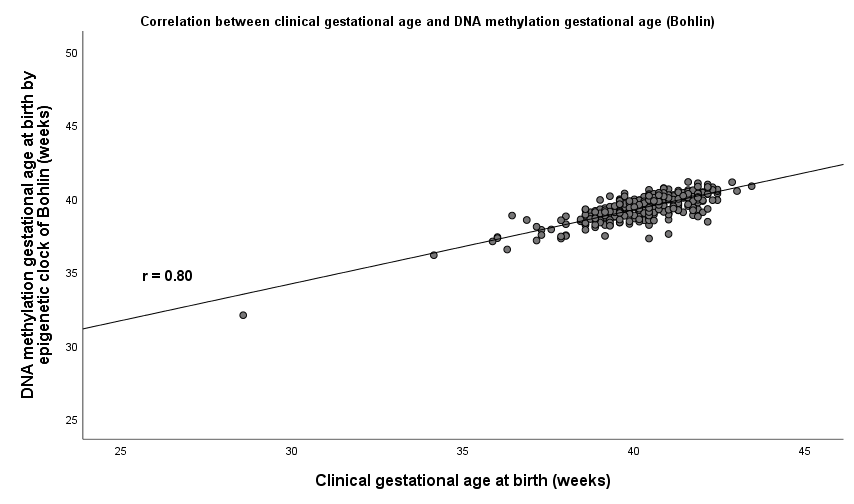


**C D**


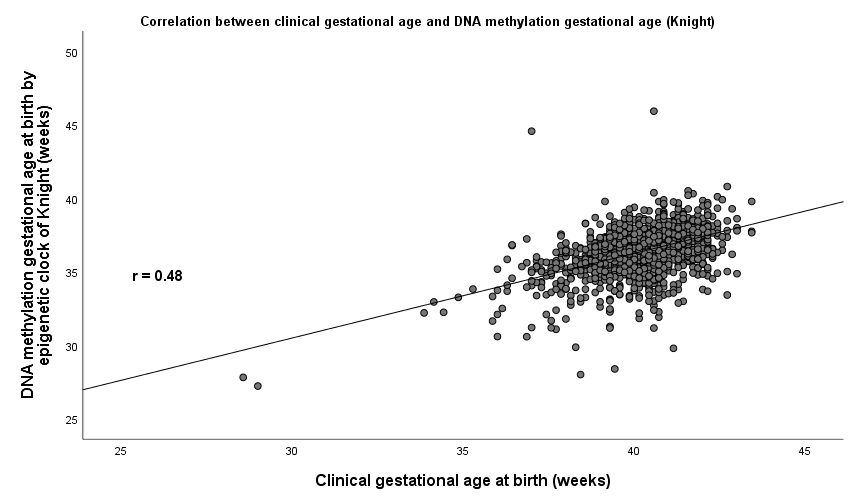

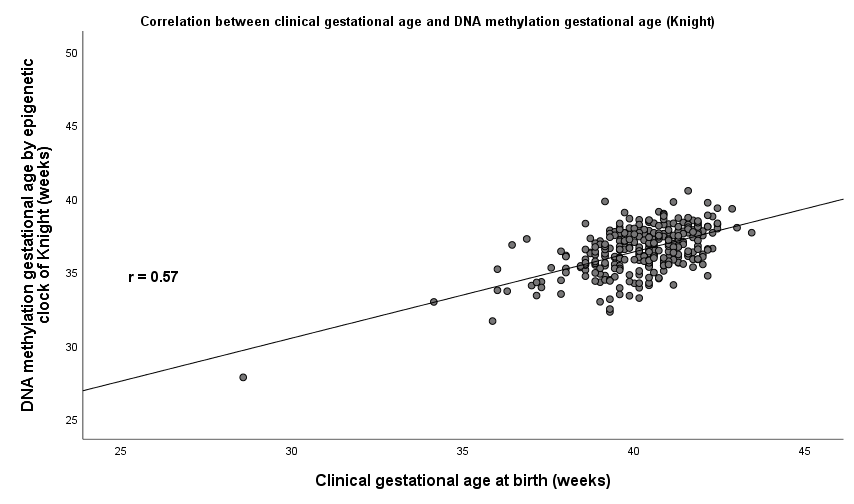


**E F**


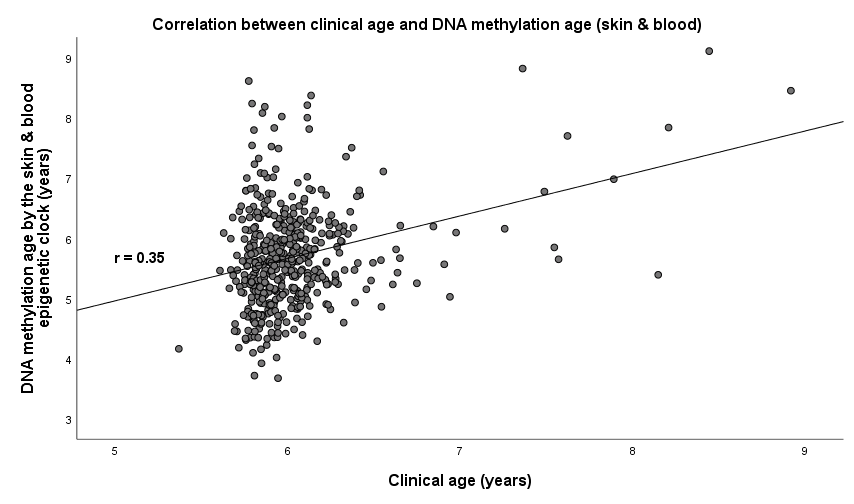

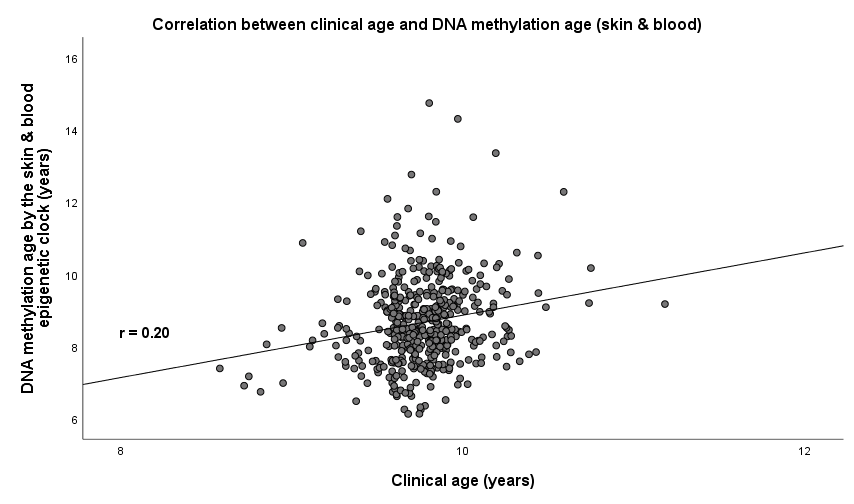
 **Figure S2. Pearson’s correlation between clinical age and DNA methylation age**

Panels A-F show the correlation between clinical age (x-axes) and DNA methylation age (y-axes), estimated by the epigenetic clocks of Bohlin or Knight (gestational age at birth), or the skin & blood epigenetic clock (age in childhood).

**A**: The correlation between clinical and DNA methylation gestational age calculated among all 1104 included children in the birth analyses using Bohlin’s clock.

**B**: The correlation between clinical and DNA methylation gestational age using Bohlin’s clock calculated among 295 children of mothers with optimal pregnancy dating based on having a regular menstrual cycle and a known first date of last menstrual period (birth analyses).

**C**: The correlation between clinical and DNA methylation gestational age calculated among all 1115 children included in the birth analyses using Knight’s clock.

**D**: The correlation between clinical and DNA methylation gestational age using Knight’s clock calculated among 297 newborns of mothers with optimal pregnancy dating based on having a regular menstrual cycle and a known first date of last menstrual period (birth analyses).

**E**: The correlation between clinical and DNA methylation age calculated among all 470 children included in the analysis at age six years using the skin & blood epigenetic clock.

**F**: The correlation between clinical and DNA methylation age calculated among all 449 children included in the analysis at age ten years using the skin & blood epigenetic clock.
