# Supplementary material for: Epigenetic age acceleration and cardiovascular outcomes in school-age children: The Generation R Study
Source: Clin Epigenetics. 2021 Nov 16;13:205. doi: 10.1186/s13148-021-01193-4 (PMC8597298; doi:10.1186/s13148-021-01193-4)
Supplement: Supplementary file 2 — Additional file 2. Table S1. Maternal and child characteristics based on imputed data (n = 1115). Table S2. Non-response analysis. Table S3. Associations of gestational age acceleration by the epigenetic clock of Bohlin with cardiovascular outcomes in children aged ten years (basic model and reduced main model). Table S4. Associations of gestational age acceleration by the epigenetic clock of Bohlin with repeated blood pressure measurements (linear mixed effect models). Table S5. Associations of gestational age acceleration by the epigenetic clock of Bohlin with carotid intima-media thickness and carotid distensibility in children aged ten years, for the right and left common carotid artery separately (main model). Table S6. Associations of gestational age acceleration by the epigenetic clock of Knight with blood pressure in children aged six years (main model). Table S7. Associations of gestational age acceleration by the epigenetic clock of Knight with cardiovascular outcomes in children aged ten years (main model). Table S8. Associations of repeated epigenetic age acceleration measurements at age 6 and 10 years with cardiovascular outcomes in children aged 10 years (conditional regression analyses) [file 13148_2021_1193_MOESM2_ESM.docx]

**Additional file 2**

**Epigenetic age acceleration and cardiovascular outcomes in school-age children: the Generation R Study**

Giulietta S. Monasso^1,2^, Vincent W.V. Jaddoe^1,2^, Leanne K. Küpers^1,2,3^, Janine F. Felix^1,2^

1. The Generation R Study Group, Erasmus MC, University Medical Center Rotterdam, Rotterdam, the Netherlands
2. Department of Pediatrics, Erasmus MC, University Medical Center Rotterdam, Rotterdam, the Netherlands
3. Division of Human Nutrition and Health, Wageningen University, Wageningen, The Netherlands

**Content: 8 Tables**

**Address for correspondence:** Janine F. Felix, MD PhD, Generation R Study Group (Na-2918), Erasmus MC, University Medical Center Rotterdam, Rotterdam, the Netherlands. Phone: +31 10 7043405, fax: +31 10 70 44645, email: j.felix@erasmusmc.nl

**Table S1. Characteristics of participating mother-child pairs (*n* 1115)^a^**

|  | **Not-imputed data** | **Imputed data^b^** |
| --- | --- | --- |
| **Maternal characteristics** |  |  |
| Age, y | 32.0 (4.0) | 32.0 (4.0) |
| Educational level |  |  |
| No, primary, secondary, *n* (%) | 353 (32.0) | 358 (32.1) |
| College or higher, *n* (%) | 749 (68.0) | 757 (67.9) |
| Pre-pregnancy body mass index, kg/m^2^ | 22.3 (18.5, 34.1) | 22.4 (18.3, 33.3) |
| Folic acid supplementation during pregnancy |  |  |
| No supplementation, *n* (%) | 62 (6.8) | 81 (7.3) |
| Started before 10 weeks, *n* (%) | 282 (31.2) | 352 (31.5) |
| Started preconception, *n* (%) | 561 (62.0) | 682 (61.2) |
| Smoking |  |  |
| Non-smoker or smoked until pregnancy was known, *n* (%) | 900 (88.5) | 985 (88.3) |
| Smoked throughout pregnancy, *n* (%) | 117 (11.5) | 130 (11.7) |
| Pregnancy dating based on last menstrual period |  |  |
| No, *n* (%) | 297 (26.6) | 297 (26.6) |
| Yes, *n* (%) | 818 (73.4) | 818 (73.4) |
| Self-reported hypertension |  |  |
| No, *n* (%) | 971 (99.1) | 1104 (99.1) |
| Yes, *n* (%) | 9 (0.9) | 10 (0.9) |
| Family history of cardiovascular disease^c^ |  |  |
| No, *n* (%) | 533 (54.2) | 611 (54.8) |
| Yes, *n* (%) | 451 (45.8) | 505 (45.2) |
| **Birth characteristics** |  |  |
| Clinical gestational age, wk | 40.2 (37.0, 42.4) | 40.2 (37.0, 42.4) |
| DNA methylation gestational age (Bohlin), wk | 39.4 (37.2, 40.8) | 39.4 (37.2, 40.8) |
| Raw gestational age acceleration (Bohlin), wk | -0.90 (-2.76, 0.92) | -0.90 (-2.76, 0.92) |
| Residual gestational age acceleration (Bohlin), wk | 0.03 (-1.24, 1.09) | 0.03 (-1.24, 1.09) |
| DNA methylation gestational age (Knight), wk | 36.5 (32.4, 39.3) | 36.5 (32.4, 39.3) |
| Raw gestational age acceleration (Knight), wk | -3.70 (-7.44, -1.07) | -3.70 (-7.44, -1.07) |
| Residual gestational age acceleration (Knight), wk | 0.15 (-3.34, 2.63) | 0.15 (-3.34, 2.63) |
| Sex |  |  |
| Boy, *n* (%) | 544 (48.8) | 544 (48.8) |
| Girl, *n* (%) | 571 (51.2) | 571 (51.2) |
| **Childhood characteristics** |  |  |
| At 6 y |  |  |
| Age at visit^d^, y | 6.0 (5.7, 7.0) | 6.0 (5.7, 7.0) |
| DNA methylation age (Skin & blood), y | 5.6 (4.3, 7.9) | 5.6 (4.3, 7.9) |
| Raw age acceleration (Skin & blood), y | -0.39 (-1.7, 1.7) | -0.4 (-1.7, 1.7) |
| Residual age acceleration (Skin & blood), y | -0.03 (-1.3, 2.1) | -0.03 (-1.3, 2.1) |
| Blood pressure, mmHg |  |  |
| Systolic | 102 (7.7) | 102 (7.7) |
| Diastolic | 60 (6.3) | 60 (6.3) |
| At 10 y |  |  |
| Age at visit, y |  |  |
| Children with DNA methylation data at birth | 9.8 (9.3, 10.5) | 9.8 (9.3, 10.5) |
| Children with DNA methylation data at 10 y^e^ | 9.8 (9.2, 10.3) | 9.8 (9.2, 10.3) |
| DNA methylation age (Skin & blood), y | 8.5 (6.7, 11.5) | 8.5 (6.7, 11.5) |
| Raw age acceleration (Skin & blood), y | -1.2 (-3.0, 1.8) | -1.2 (-3.0, 1.8) |
| Residual age acceleration (Skin & blood), y | -0.10 (-1.9, 2.8) | -0.10 (-1.9, 2.8) |
| Blood pressure, mmHg |  |  |
| Systolic | 103 (7.7) | 103 (7.7) |
| Diastolic | 58 (6.2) | 58 (6.2) |
| Common carotid artery intima-media thickness, mm | 0.45 (0.04) | 0.45 (0.04) |
| Common carotid artery distensibility^f^, kPa^-1^*10^-3^ | 56.0 (37.3, 85.0) | 56.0 (37.3, 85.0) |

a For the analyses based on Bohlin’s epigenetic clock, we excluded 11 newborns with missing values for some of the required CpGs, leaving 1104 children for analysis in the full population and 295 children in the subgroup with optimal pregnancy dating. Values are based on imputed data and are mean (SD) or median (95% range) for continuous variables and numbers (%) for categorical variables. Exposures and outcomes were not imputed, as well as clinical gestational age and method of pregnancy dating.

b Missing data: maternal education: n=13; maternal body mass index: n=170; folic acid supplementation: n=210; maternal smoking: n=98; maternal self-reported hypertension: n=135; family history of cardiovascular disease: n=131.

c We obtained this information from maternal questionnaires sent out during pregnancy. We created one variable for family history of cardiovascular disease, defined as a first-degree relative with any of hypertension, myocardial infarction below the age of 65, cerebrovascular accident.

d Of these 470 children, 12 children were not included in the analyses at birth, as they had no cord blood DNA methylation measured.

e Of these 449 children, 14 children were not included in the analyses at birth, as they had no cord blood DNA methylation measured.

f Indicate values before natural-log transformation.

**Table S2. Non-response analysis comparing children with DNA methylation data available, with/without cardiovascular outcome available^a,b^**

|  | **Included**  **n = 1115** | **Not included**  **n = 281** | **P value^c^** |
| --- | --- | --- | --- |
| **Maternal characteristics** |  |  |  |
| Age, y | 32.0 (4.0) | 30.7 (4.8) | < 0.001 |
| Educational level |  |  | < 0.001 |
| No, primary, secondary, *n* (%) | 353 (32.0) | 125 (45.6) |  |
| College or higher, *n* (%) | 749 (68.0) | 149 (54.4) |  |
| Pre-pregnancy body mass index, kg/m^2^ | 22.3 (18.5, 34.1) | 22.1 (18.1, 33.3) | 0.99 |
| Folic acid supplementation during pregnancy |  |  | <0.001 |
| No supplementation, *n* (%) | 62 (6.8) | 38 (16.2) |  |
| Started before 10 weeks, *n* (%) | 282 (31.2) | 70 (29.8) |  |
| Started preconception, *n* (%) | 561 (62.0) | 127 (54.0) |  |
| Smoking |  |  | <0.001 |
| Non-smoker or smoked until pregnancy was known, *n* (%) | 900 (88.5) | 196 (74.5) |  |
| Smoked throughout pregnancy, *n* (%) | 117 (11.5) | 67 (25.5) |  |
| Pregnancy dating based on last menstrual period |  |  | 0.06 |
| No, *n* (%) | 297 (26.6) | 290 (67.6) |  |
| Yes, *n* (%) | 818 (73.4) | 91 (32.4) |  |
| **Birth characteristics** |  |  |  |
| Clinical gestational age, wk | 40.2 (37.0, 42.4) | 40.3 (35.9, 42.3) | 0.18 |
| DNA methylation gestational age (Bohlin), wk | 39.4 (37.2, 40.8) | 39.5 (36.2, 40.8) | 0.98 |
| Raw gestational age acceleration (Bohlin), wk | -0.9 (-2.8, 0.9) | -0.8 (-2.3, 1.3) | 0.09 |
| DNA methylation gestational age (Knight), wk | 36.5 (32.4, 39.3) | 36.4 (31.7, 39.0) | 0.13 |
| Raw gestational age acceleration (Knight), wk | -3.7 (-7.4, -1.1) | -3.8 (-7.4, -1.4) | 0.73 |
| Sex |  |  | 0.004 |
| Boy, *n* (%) | 544 (48.8) | 164 (58.4) |  |
| Girl, *n* (%) | 571 (51.2) | 117 (41.6) |  |
| **Childhood characteristics** |  |  |  |
| Age at visit age 10 y, y | 9.8 (9.3, 10.5) | 9.7 (9.1, 11.4) | 0.10 |

a The non-response analysis compared the 1115 children who were included in the analyses at birth to those who had information on DNA methylation data at birth available, but were not included (n=325) because information on all cardiovascular outcomes was missing (=269), or their sibling was included in the analyses (n=12).

b Values are based on non-imputed data and are mean (SD) or median (95% range) for continuous variables and numbers (%) for categorical variables.

c P values for differences in subject characteristics between groups were calculated performing independent sample t-tests (normally distributed continuous variables), Mann Whitney tests (not normally distributed continuous variables) and chi-square tests (categorical variables).

Table S3. Associations of gestational age acceleration by the epigenetic clock of Bohlin with cardiovascular outcomes in children aged ten years (basic model and reduced main model)^a,b^

|  | **Systolic blood pressure**  ***n*=1097** | | **Diastolic blood pressure**  ***n*=1098** | | | **Common carotid artery**  **intima-media thickness**  ***n*=1060** | | | **Common carotid artery**  **distensibility**  ***n*=943** | | |  |
| --- | --- | --- | --- | --- | --- | --- | --- | --- | --- | --- | --- | --- |
|  | **Difference**  **(95% CI) in SDS** | **P value** | | **Difference**  **(95% CI) in SDS** | **P value** | | **Difference**  **(95% CI) in SDS** | **P value** | | **Difference**  **(95% CI) in SDS** | **P value** | |
| Basic model |  |  | |  |  | |  |  | |  |  | |
| Raw | 0.056 (-0.01, 0.12) | 0.09 | | 0.035 (-0.03, 0.10) | 0.30 | | -0.004 (-0.07, 0.06) | 0.91 | | -0.005 (-0.08, 0.07) | 0.88 | |
| Residual | -0.012 (-012, 0.10) | 0.83 | | 0.027 (-0.09, 0.14) | 0.64 | | 0.030 (-0.08, 0.14) | 0.59 | | 0.06 (-0.05, 0.18) | 0.29 | |
| Reduced main model |  |  | |  |  | |  |  | |  |  | |
| Raw | 0.034 (-0.03, 0.10) | 0.30 | | 0.020 (-0.05, 0.09) | 0.54 | | -0.001 (-0.07, 0.06) | 0.99 | | -0.002 (-0.07, 0.07) | 0.96 | |
| Residual | -0.047 (-0.15, 0.05) | 0.36 | | -0.009 (-0.11, 0.09) | 0.86 | | 0.002 (-0.10, 0.11) | 0.97 | | 0.048 (-0.06, 0.16) | 0.39 | |

CI, confidence interval; SDS, standard deviation score.

Values represent regression coefficients (95% confidence interval) and reflect the difference in cardiovascular outcome in SDS per change in raw and residual gestational age acceleration (in weeks) at birth. Shown results are based on the basic model and reduced main model. The basic model was adjusted for child sex, batch effects in DNA methylation data (by including sample plate number), child age at outcome measurement and cell types. The reduced main model was adjusted for child sex, batch effects (by including plate number), child age at outcome measurement and maternal confounders (age, education, pre-pregnancy body mass index and folic acid supplementation and smoking during pregnancy), but not corrected for cell type proportion.

a For the analyses based on Bohlin’s epigenetic clock, we excluded 11 of the 1115 included newborns with missing values for some of the required CpGs, leaving 1104 children for analysis in the full population.

b Raw gestational age acceleration was obtained by subtracting the clinical estimate of gestational age from DNA methylation gestational age. Residual gestational age acceleration was calculated from the residuals from a regression model of DNA methylation gestational age on clinical gestational age.

Table S4. Associations of gestational age acceleration by the epigenetic clock of Bohlin with repeated blood pressure measurements (linear mixed effect models)^a,b^

|  | **Systolic blood pressure**  **n=994** | | | **Diastolic blood pressure**  **n=994** | | |
| --- | --- | --- | --- | --- | --- | --- |
|  | **Difference (95% CI) in SDS** | **P value** | **P value for interaction with age** | **Difference (95% CI) in SDS** | **P value** | **P value for interaction with age** |
| Raw | 0.033 (-0.11 0.17) | 0.64 | 0.99 | -0.059 (-0.22, 0.10) | 0.47 | 0.40 |
| Residual | 0.010 (-0.13, 0.15) | 0.89 | 0.72 | -0.064 (-0.22, 0.09) | 0.43 | 0.48 |

CI, confidence interval; SDS, standard deviation score.

Main effects (95% confidence interval), p-values and p-values for interaction with age at blood pressure measurement obtained from linear mixed effects models. Main effects reflect the difference in blood pressure measurement in SDS for an SDS change in raw or residual gestational age acceleration (in weeks) at birth. Models include an interaction term between gestational age acceleration and child age at blood pressure measurement and a random intercept for each child. To decrease the number of covariates in the models, we first obtained standardized residuals for both raw and residual epigenetic gestational age acceleration from the regression of epigenetic gestational age acceleration on batch and all cell types and used these residuals as the exposure. The models were additionally adjusted for child sex and maternal confounders (age, education, pre-pregnancy body mass index and folic acid supplementation and smoking during pregnancy).

a For the analyses based on Bohlin’s epigenetic clock, we excluded 11 of the 1115 included newborns with missing values for some of the required CpGs, leaving 1104 children for analysis in the full population. Of these, 994 children had information on blood pressure available.

b Raw gestational age acceleration was obtained by subtracting the clinical estimate of gestational age from DNA methylation gestational age. Residual gestational age acceleration was calculated from the residuals from a regression model of DNA methylation gestational age on clinical gestational age.

**Table S5. Associations of gestational age acceleration by the epigenetic clock of Bohlin with carotid intima-media thickness and carotid distensibility in children aged ten years, for the right and left common carotid artery separately (main model)^a,b^**

|  | **Common carotid artery intima-media thickness** | | | | **Common carotid artery distensibility** | | | |
| --- | --- | --- | --- | --- | --- | --- | --- | --- |
|  | **Right side *n*=1040** | | **Left side *n=*1058** | | **Right side *n=* 930** | | **Left side *n=*940** | |
|  | **Difference**  **(95% CI) in SDS** | **P value** | **Difference**  **(95% CI) in SDS** | **P value** | **Difference**  **(95% CI) in SDS** | **P value** | **Difference**  **(95% CI) in SDS** | **P value** |
| Raw | 0.019 (-0.05, 0.09) | 0.57 | -0.018 (-0.09, 0.05) | 0.60 | -0.008 (-0.08, 0.06) | 0.82 | 0.022 (-0.05, 0.09) | 0.55 |
| Residual | 0.042 (-0.07, 0.15) | 0.46 | 0.013 (-0.10, 0.13) | 0.82 | 0.080 (-0.04, 0.20) | 0.19 | 0.042 (-0.08, 0.16) | 0.49 |

CI, confidence interval; SDS, standard deviation score.

Values represent regression coefficients (95% confidence interval) and reflect the difference in cIMT and distensibility in SDS per change in raw and residual gestational age acceleration (in weeks) at birth. Shown results are based on the main model which was adjusted for child sex, batch effects in DNA methylation data (by including sample plate number), child age at outcome measurement, cell types and maternal confounders (age, education, pre-pregnancy body mass index and folic acid supplementation and smoking during pregnancy).

a For the analyses based on Bohlin’s epigenetic clock, we excluded 11 newborns with missing values for some of the required CpGs, leaving 1104 children for analysis in the full population.

b Raw gestational age acceleration was obtained by subtracting the clinical estimate of gestational age from DNA methylation gestational age. Residual gestational age acceleration was calculated from the residuals from a regression model of DNA methylation gestational age on clinical gestational age.

Table S6. Associations of gestational age acceleration by the epigenetic clock of Knight with blood pressure in children aged six years (main model)^a^

|  | **Systolic blood pressure** | | | **Diastolic blood pressure** | |
| --- | --- | --- | --- | --- | --- |
|  | **Difference (95% CI) in SDS** | **P value** | **Difference (95% CI) in SDS** | | **P value** |
| Full population (n=1115) | *n*=1005 |  | *n*=1005 | |  |
| Raw | 0.033 (-0.01, 0.08) | 0.14 | -0.005 (-0.05, 0.03) | | 0.82 |
| Residual | 0.020 (-0.03, 0.07) | 0.45 | -0.011 (-0.06, 0.04) | | 0.67 |
| Subgroup: optimal pregnancy dating (n=297) | *n*=266 |  | *n*=266 | |  |
| Raw | 0.003 (-0.10, 0.11) | 0.96 | -0.041 (-0.14, 0.06) | | 0.44 |
| Residual | -0.005 (-0.13, 0.12) | 0.93 | -0.024 (-0.15, 0.10) | | 0.70 |

CI, confidence interval; SDS, standard deviation score.

Values represent regression coefficients (95% confidence interval) and reflect the difference in blood pressure in SDS per change in raw and residual gestational age acceleration (in weeks) at birth. Shown results are based on the main model which was adjusted for child sex, batch effects in DNA methylation data (by including sample plate number), child age at outcome measurement, cell types and maternal confounders (age, education, pre-pregnancy body mass index and folic acid supplementation and smoking during pregnancy).

a Raw gestational age acceleration was obtained by subtracting the clinical estimate of gestational age from DNA methylation gestational age. Residual gestational age acceleration was calculated from the residuals from a regression model of DNA methylation gestational age on clinical gestational age.

**Table S7. Associations of gestational age acceleration by the epigenetic clock of Knight with cardiovascular outcomes in children aged ten years (main model)^a^**

|  | **Systolic blood pressure** | | **Diastolic blood pressure** | | **Common carotid artery**  **intima-media thickness** | | **Common carotid artery**  **Distensibility** | |  |
| --- | --- | --- | --- | --- | --- | --- | --- | --- | --- |
|  | **Difference**  **(95% CI) in SDS** | **P value** | **Difference**  **(95% CI) in SDS** | **P value** | **Difference**  **(95% CI) in SDS** | **P value** | **Difference**  **(95% CI) in SDS** | **P value** | |
| Full population (n=1115) | *n*=1108 |  | *n*=1109 |  | *n*=1071 |  | *n*=954 |  | |
| Raw | 0.031 (-0.01, 0.07) | 0.14 | 0.030 (-0.01, 0.07) | 0.16 | -0.006 (-0.05, 0.04) | 0.78 | 0.002 (-0.04, 0.05) | 0.92 | |
| Residual | 0.017 (-0.03, 0.06) | 0.48 | 0.030 (-0.02, 0.08) | 0.22 | -0.004 (-0.05, 0.04) | 0.87 | 0.014 (-0.04, 0.07) | 0.60 | |
| Subgroup: optimal pregnancy dating (n=297) | *n*=295 |  | *n*=295 |  | *n*=282 |  | *n*=257 |  | |
| Raw | 0.045 (-0.06, 0.14) | 0.38 | 0.059 (-0.04, 0.16) | 0.26 | 0.011 (-0.09, 0.11) | 0.83 | 0.005 (-0.10, 0.11) | 0.94 | |
| Residual | -0.007 (-0.12, 0.11) | 0.91 | 0.052 (-0.07, 0.17) | 0.39 | -0.005 (-0.12, 0.11) | 0.93 | 0.074 (-0.049, 0.20) | 0.24 | |

CI, confidence interval; SDS, standard deviation score.

Values represent regression coefficients (95% confidence interval) and reflect the difference in cardiovascular outcome in SDS per change in raw and residual gestational age acceleration (in weeks) at birth. Shown results are based on the main model which was adjusted for child sex, batch effects in DNA methylation data (by including sample plate number), child age at outcome measurement, cell types and maternal confounders (age, education, pre-pregnancy body mass index and folic acid supplementation and smoking during pregnancy).

a Raw gestational age acceleration was obtained by subtracting the clinical estimate of gestational age from DNA methylation gestational age. Residual gestational age acceleration was calculated from the residuals from a regression model of DNA methylation gestational age on clinical gestational age.

**Table S8. Associations of repeated epigenetic age acceleration measurements at age 6 and 10 years with cardiovascular outcomes in children aged 10 years (conditional regression analyses)^a^**

|  |  | **Standard Deviation Scores, Regression Coefficients (95% CI)** | | | | | | | | | | |
| --- | --- | --- | --- | --- | --- | --- | --- | --- | --- | --- | --- | --- |
|  |  | **Systolic blood pressure**  **n=329** | | | **Diastolic blood pressure**  **n=329** | | | **Common carotid artery intima-media thickness**  **n=317** | | | **Common carotid artery distensibility**  **n=289** | |
|  |  | **Difference**  **(95% CI) in SDS** | **P value** | **Difference**  **(95% CI) in SDS** | | **P value** | **Difference**  **(95% CI) in SDS** | | **P value** | **Difference**  **(95% CI) in SDS** | | **P value** |
| **Raw** |  |  |  |  | |  |  | |  |  | |  |
| 6 years, SDS |  | -0.021 (-0.13, 0.09) | 0.71 | 0.019 (-0.09, 0.13) | | 0.73 | -0.074 (-0.18, 0.03) | | 0.18 | -0.021 (-0.13, 0.09) | | 0.72 |
| 10 years, SDS |  | 0.012 (-0.10, 0.12) | 0.83 | 0.053 (-0.05, 0.16) | | 0.33 | -0.014 (-0.12, 0.09) | | 0.79 | -0.067 (-0.19, 0.05) | | 0.27 |
| **Residual** |  |  |  |  | |  |  | |  |  | |  |
| 6 years, SDS |  | -0.020 (-0.13 ,0.09) | 0.72 | 0.026 (-0.08, 0.13) | | 0.64 | -0.067 (-0.18, 0.04) | | 0.22 | -0.028 (-0.14, 0.09) | | 0.63 |
| 10 years, SDS |  | 0.013 (-0.10, 0.12) | 0.82 | 0.052 (-0.06, 0.16) | | 0.34 | -0.018 (-0.13, 0.09) | | 0.74 | -0.064 (-0.18, 0.06) | | 0.29 |

Abbreviation: CI, confidence interval

a Values represent regression coefficients (95% confidence interval) from conditional regression analyses and reflect the difference in systolic or diastolic blood pressure, carotid intima-media thickness and log-transformed carotid distensibility in SDS per change in raw and residual age acceleration (in weeks). Included children had information on gestational age acceleration at both ages, which were assessed independently of each other. To decrease the number of covariates in the models, at both age 6 and 10 years, we first obtained standardized residuals for both raw and residual epigenetic age acceleration from the regression of epigenetic age acceleration on batch and all cell types and used these residuals as the exposure. The models were additionally adjusted for child sex and age at outcome measurement and maternal confounders (age, education, pre-pregnancy body mass index and folic acid supplementation and smoking during pregnancy).
